# Temperature overrides nutritional cues for optimal oviposition decision in a polyphagous invasive insect

**DOI:** 10.1242/jeb.251743

**Published:** 2026-02-12

**Authors:** Wendy Destierdt, Gwenaëlle Deconninck, José E. Crespo, Esteban Moyer, Vincent Foray, Olivier Chabrerie, Sylvain Pincebourde

**Affiliations:** ^1^ Institut de Recherche sur la Biologie de l'Insecte, UMR 7261, CNRS - Université de Tours, 37200 Tours, France; ^2^ Biodiversity Research Centre, Earth and Life Institute, UCLouvain, B-1348 Louvain-la-Neuve, Belgium; ^3^ Department of Biology, Lund University, 223 62 Lund, Sweden; ^4^ Laboratorio de Entomología Experimental - Grupo de Ecología Térmica de Insectos (LEE-GETI), Departamento de Ecología, Genética y Evolución, Facultad de Ciencias Exactas y Naturales, Instituto IEGEBA (CONICET-UBA), Universidad de Buenos Aires, Buenos Aires C1428EGA, Argentina; ^5^ EDYSAN, Ecologie et Dynamique des Systèmes Anthropisés, UMR 7058 CNRS, Université de Picardie Jules Verne, 80025 Amiens Cedex 1, France

**Keywords:** *Drosophila suzukii*, Polyphagy, Decision making, Preference–performance hypothesis, Oviposition choice, *Wolbachia*

## Abstract

Polyphagous insects rely on multiple cues to choose oviposition sites, including substrate temperature and nutritional quality, which often do not coincide. We examined how females of the invasive fly *Drosophila suzukii* make oviposition decisions when temperature and nutrition mismatch, and whether infection with the symbiotic bacterium *Wolbachia* influences these choices. We first quantified female performance (egg number, offspring development time, survival and mass) on four fruit purees at three ambient temperatures. We then assessed oviposition preferences when either substrate temperature or fruit quality varied independently. Finally, we conducted multi-choice experiments combining thermal and nutritional cues to test which most strongly drives oviposition. Both temperature and fruit quality affected offspring performance. Although females did not always choose the most favourable fruit, they consistently prioritised thermally optimal sites, even when these were nutritionally suboptimal. This behaviour gave partial support to the preference–performance hypothesis, which mainly held for temperature – the factor with the strongest effect on offspring development and survival in no-choice tests. *Wolbachia* infection enhanced offspring survival and reduced development time. It also altered oviposition patterns, leading to a more even distribution of eggs across fruit, though females maintained their preference for thermally favourable sites. Our findings suggest that the invasive success of *D. suzukii* could partly result from its capacity to select oviposition sites that maximise offspring performance under variable conditions. More broadly, they highlight the need to study behavioural decisions under conflicting environmental constraints to understand how behavioural flexibility contributes to individual fitness and population persistence in changing environments.

## Teaser

**Highlighted Article:** A polyphagous insect makes optimal oviposition decisions when facing contradicting cues (temperature and nutrition), corroborating the preference–performance hypothesis in complex scenarios.

## ADDITIONAL LANGUAGE ABSTRACT (FRENCH)

Les insectes polyphages s'appuient sur de nombreux signaux pour choisir leurs sites de ponte, notamment la température du substrat et sa qualité nutritionnelle, qui ne coïncident pas toujours. Cette étude analyse le choix de ponte des femelles de la mouche invasive *Drosophila suzukii* lorsque ces signaux sont contradictoires, ainsi que l'effet de l'infection par la bactérie symbiotique *Wolbachia*. L'étude s'est déroulée en quatre étapes: (1) la mesure des performances des descendants (nombre d'œufs, temps de développement, survie, masse de la progéniture) sur quatre purées de fruits à trois températures ambiantes; l'évaluation des préférences de ponte lorsque (2) la qualité du fruit ou (3) la température du substrat variaient indépendamment; et (4) des tests à choix multiples pour déterminer le signal influençant le plus le choix de ponte. La température et la qualité du fruit impactent toutes deux la performance de la descendance. Alors que les femelles ne choisissent pas toujours le fruit le plus favorable, elles privilégient systématiquement les sites les plus chauds, thermiquement optimaux, même lorsqu'ils sont nutritionnellement sous-optimaux. Ce comportement soutient l'hypothèse de préférence–performance, la température étant le principal facteur déterminant pour le développement et la survie. L'infection par *Wolbachia* augmente la survie et réduit le temps de développement, tout en modifiant les choix de ponte avec une répartition plus homogène entre les fruits, sans altérer la préférence pour les sites chauds. Ces résultats suggèrent que le succès invasif de *D. suzukii* pourrait découler de sa capacité à sélectionner des sites de ponte maximisant la performance de la descendance en conditions environnementales variables. Ils soulignent également l'importance d'étudier les décisions comportementales face à des contraintes antagonistes pour comprendre la contribution de la flexibilité comportementale à la valeur sélective des individus et à la persistance des populations.

## INTRODUCTION

Oviposition site selection is a key determinant of female fitness in many animals including phytophagous insects (Gripenberg et al., 2010). Oviposition strategies require females to assess and sort among potentially conflicting cues, including nutritional resource quality or the probability of finding a suitable resource (Scheirs et al., 2000; Janz, 2003; García-Robledo et al., 2010). The preference–performance hypothesis (PPH), also known as ‘mother knows best’, predicts that females maximise their fitness by laying eggs preferentially on substrates that maximise their offspring performance (such as offspring survival rate, development time and size; Gripenberg et al., 2010). However, most studies investigating this hypothesis only considered the nutritional aspect of the substrate (e.g. host plant), neglecting other abiotic factors such as temperature of the ovipositing substrate (reviewed in Gripenberg et al., 2010; Charlery de la Masselière et al., 2017).

Females appear to influence the success of egg hatching and development by selecting oviposition sites with favourable microclimates (Feder et al., 2000; Stillwell and Fox, 2005). The consequences of this choice on larval performance (i.e. development rate, survival) can be amplified when larvae are unable to move between microhabitats (Scheirs et al., 2000; García-Robledo et al., 2010). In polyphagous (i.e. generalists) insects that oviposit in fruit, offspring performance is influenced by both fruit microclimate and nutritional quality (Janz, 2003), which could lead to complex trade-offs. However, close associations with microorganisms, such as *Wolbachia* (Zug and Hammerstein, 2015), can enable insects to feed efficiently on a wider variety of plants (Hosokawa et al., 2010; Sugio et al., 2015; Giron et al., 2018; Benhamou et al., 2021) by providing digestive enzymes, producing vitamins and/or detoxifying plant defence compounds (Dillon and Dillon, 2004; Engel and Moran, 2013; Ashra and Nair, 2022). Females infected by *Wolbachia* may therefore have different oviposition preferences than uninfected females.

*Drosophila suzukii* (Diptera: Drosophilidae) is an interesting model with which to study the interplay between temperature, host-plant quality and *Wolbachia* infection in shaping oviposition choice. This invasive species, native to Southeast Asia, is highly polyphagous, infesting a wide range of cultivated and wild fruit (Lee et al., 2015; Poyet et al., 2015). The broad dietary spectrum of *D. suzukii*, along with the extensive variety of host plants available year-round, ensures a constant supply of trophic resources contributing to its persistence and invasive success (Poyet et al., 2015; Deconninck et al., 2024a,b, 2025a). However, its polyphagous nature exposes the fly to diets that vary in quality, which can influence population dynamics (Poyet et al., 2015; Olazcuaga et al., 2019; Larges et al., 2025). *Drosophila suzukii* can also be infected by the endosymbiotic bacteria *Wolbachia*, which has been shown to improve the fitness of offspring after a diet shift (Deconninck et al., 2024c).

**Fig. 1.**
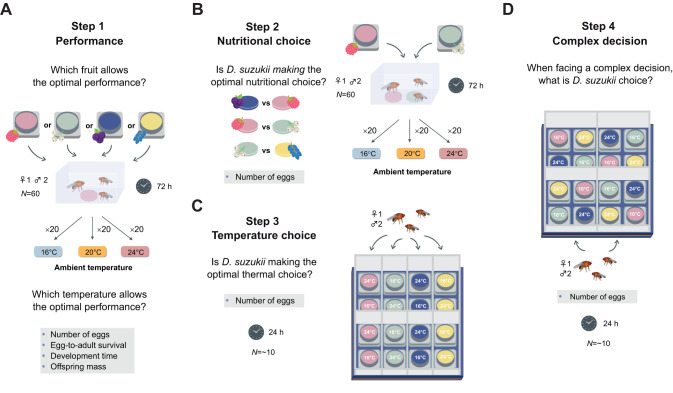
**Experimental design.** (A) Step 1: performance in no choice experiments. (B) Step 2: fruit-based oviposition choice. (C) Step 3: temperature-based oviposition choice. (D) Step 4: optimal decision making.

The present study aimed to explore the interplay between host-plant quality and temperature in shaping oviposition site selection in *D. suzukii*, and whether *Wolbachia* infection modulates the choice of oviposition site. We followed four steps (Fig. 1). The first assessed *D. suzukii*'s performance on different fruit in no-choice experiments and across a range of temperatures. We used fruit purees from four common host-plant species (*Mahonia japonica*, *Ribes nigrum*, *Rubus idaeus* and *Viscum album*) on which the performance was expected to differ (Poyet et al., 2015; Olazcuaga et al., 2019; Deconninck et al., 2025a; Larges et al., 2025). The second step evaluated fruit-based oviposition choices under different ambient temperatures. We hypothesised that females choose oviposition sites based on optimal nutritional value at any temperature, in line with the PPH (Gripenberg et al., 2010). The third step investigated female oviposition choices based on substrate temperature for each fruit at constant air temperature. We predicted that females consistently select oviposition sites based on the most favourable substrate temperature for larval development, with a preference for warmer temperatures close to their optimum (Raynaud-Berton et al., 2024). These three steps allowed us to predict which combination of fruit and temperature are optimal for offspring fitness. Based on these predictions, the final step investigated the existence of an optimal decision making by confronting females to a combination of sub-optimal fruit at optimal substrate temperature and vice versa, expecting that the factor with the highest effect on offspring fitness was prioritised by the female. For all these steps, we hypothesised that the presence of *Wolbachia* would improve offspring performance (Deconninck et al., 2024c) and could influence the females' oviposition choice with a preference towards colder substrates (Truitt et al., 2019).

## MATERIALS AND METHODS

### Insect strain and laboratory maintenance

The *Drosophila suzukii* (Matsumura 1931) stock used in this experiment has been described in detail in Deconninck et al. (2024c). Briefly, the flies were collected from infested raspberries in 2020 in Rennes, France (48°7′2.158″N, 1°40′40.053″W), by ECOBIO Laboratory (Rennes). Two lines differing by the presence (W+) or absence (W−) of *Wolbachia* (*w*Suz) were obtained with tetracycline treatment (method by Hague et al., 2021). To reduce confounding effects of the antibiotic treatment, the W– fly microbiota was restored by rearing W– flies on a substrate inoculated with the faeces of W+ flies for three generations (method by Strunov et al., 2022; used in Deconninck et al., 2024c). Before and after experiments, *Wolbachia* presence in the two lines was assessed by diagnostic PCR using *Wolbachia*-specific primers targeting the *wsp* gene (81F/691R) (Braig et al., 1998). The flies were reared in 100 ml plastic bottles containing ∼30 ml of standard cornmeal diet and incubated at 20°C, under a 12 h:12 h light:dark cycle and 75% relative humidity (Strader, EV1300, Angers, France). At least 30 bottles with 50–200 flies per line were used for continuous maintenance. In all experiments, W– and W+ flies aged between 10 and 15 days were used.

### Artificial fruit media

Purees were made from ripe fruit collected in the wild or originating from growers: *Viscum album* and *Mahonia japonica* were collected in Tours, France (47°23′24.171″N, 0°41′20.136″E), *Ribes nigrum* originated from Anjou, France (Le Domaine du Framboisier, 48°58′44.196″N, 0°46′57.67″E), and organic *Rubus idaeus* fruit were bought (Maison Thiriet, fruit origin Serbia). Fruit was frozen (–20°C) before experiments. The fruit purees were prepared using a recipe adapted from Olazcuaga et al. (2019): each fruit (600 g) was mixed with agar (15 g) dissolved in warm water (1 litre), inactive yeast (65 g) and nipagin (4 g) dissolved in 70% ethanol (10 ml). Mistletoe purees were coloured pink with red food colourant to minimise the influence of colour on egg-laying choice (Deconninck et al., 2024c). To preserve their flavours and colours, the purees were stored at 4°C until use the following day. Fruit purees were used instead of whole fruit to isolate the effects of fruit nutritional composition on preference and performance from variables such as skin thickness, shape, colour and size (Lee et al., 2016).

### Thermal landscape

We designed a thermal landscape simulator (TLS; Fig. S1) that allowed the control of substrate temperature (i.e. egg microenvironment) while keeping constant the ambient air temperature (i.e. adult microenvironment). It consisted of 16 aluminium plates that were warmed (24°C) or cooled (16°C) independently by Peltier elements (45 W, Quick-cool^®^, Germany) and on which 3D-printed egg-laying cylinders containing fruit puree were placed (Fig. S1). The temperature was set using Arduino temperature controllers (UNO R3). A water-cooling system ran continuously below the Peltier elements to prevent overheating of the TLS. Arenas enclosing two or four plates were created with separation grids covered with mesh that allowed the flies to move freely and isolated replicates. Prior to the experiment, temperatures were calibrated using thermocouples (T-type copper Constantan, diameter 0.2 mm, TC Direct, Dardilly, France) inserted in egg-laying cylinders filled with fruit puree and placed on each Peltier module (Fig. S2). A piece of absorbent paper was put in the fruit purees to prevent condensation. The TLS was installed in a laboratory room set at 20°C with a 12 h:12 h light:dark cycle.

### Step 1: Performance in no-choice experiments

To assess the performance (i.e. number of eggs laid, development time, survival and mass of offspring) of *D. suzukii* on each fruit puree, we used egg-laying boxes made of crystal polystyrene (60×45×50 mm) with a hole to accommodate one 3D-printed egg-laying cylinder (6×30 mm) containing 5 ml of fruit puree. In each box, one female and two males were introduced. The boxes were incubated at 16°C, 20°C or 24°C with a 12 h:12 h light:dark cycle and 75% relative humidity (Strader, EV1300). Flies were removed after 3 days and eggs were counted under a stereomicroscope (Leica MZ125, magnification up to ×10). We considered the number of eggs laid as a proxy of oviposition performance. Even if the number of eggs laid in the substrate could also be influenced by the female decision to withhold eggs (Yang et al., 2008; Soto et al., 2012), especially on low quality substrates, withholding would still induce a reduction in fitness. Development was monitored daily, tracking the number of pupae and emerging adult flies until they all hatched. Survival rate and developmental time were calculated for each stage: egg-to-pupa, pupa-to-adult and egg-to-adult. Upon emergence, adult flies were sexed and weighed using a laboratory precision balance (0.01 mg; QUINTIX125D-1S; Sartorius, Göttingen, Germany). Each treatment had 20 replicates.

### Step 2: Fruit-based oviposition choice

To assess female oviposition preference for fruit, we used polystyrene boxes (90×60×50 mm) with two egg-laying cylinders containing 5 ml of fruit puree. We tested two pairs of fruit with contrasting performance (i.e. *V. album* versus *M. japonica* and *R. idaeus* versus *R. nigrum*) and one pair with high performance (i.e. *V. album* versus *R. idaeus*), according to step 1. In each box, one female and two males were introduced. Boxes were randomly placed within climatic chambers set at 16°C, 20°C or 24°C with a 12 h:12 h light:dark cycle and 75% relative humidity (Strader, EV1300). After 3 days, the number of eggs laid in each cylinder was counted under a stereomicroscope. Each treatment had 20 replicates.

### Step 3: Temperature-based oviposition choice

To assess female oviposition preference for substrate temperature, we divided the TLS into eight sections in which two egg-laying cylinders containing 5 ml of fruit puree from a single fruit (i.e. *M. japonica*, *R. ideaus*, *R. nigrum* or *V. album*) were placed and maintained at 16°C and 24°C. Air temperature within the sections was maintained at 20–22°C, ensuring that any preference was linked to substrate temperature rather than to the air temperature experienced by the female. One female and two males were introduced into each section and, after 24 h, the number of eggs laid in each fruit puree was counted under a stereomicroscope. Each treatment had at least 10 replicates.

### Step 4: Complex decision making

To assess the influence of fruit and substrate temperature on *D. suzukii* oviposition preference, we divided the TLS into four sections, each section exposing one female and two males to four egg-laying cylinders with the nutritionally optimal fruit purees (*V. album* and *R. idaeus*) maintained at suboptimal temperature (16°C) and the suboptimal fruit purees (*M. japonica* and *R. nigrum*) maintained at optimal temperature (24°C). This setup was based on offspring performance in the previous steps (see Results). After 24 h, the number of eggs laid in each egg-laying cylinder was counted. Each treatment had 20 replicates.

### Statistical analyses

Data on performance (step 1) were analysed using generalised linear mixed models (GLMMs): the total number of eggs laid was analysed using GLMMs with negative binomial error distribution and log link (R package ‘glmmTMB’; Brooks et al., 2017), offspring survival (alive=1, dead=0) was analysed using GLMMs with binomial distribution and logit link (R package ‘lme4’; Bates et al., 2015), mean development time was analysed using GLMMs on log-transformed data (R package ‘lme4’; Bates et al., 2015) and offspring mass was analysed using GLMMs on square-root-transformed data (R package ‘lme4’; Bates et al., 2015). Temperature was included as a continuous predictor in these GLMMs, and fruit puree and *Wolbachia* infection were included as fixed categorical effects. All interactions among these predictors were tested, and female ID was included as a random effect. For development time and mass, temperature was modelled using a second-degree polynomial (quadratic) term, as this significantly improved model fit based on Akaike's information criterion (AIC), Bayesian information criterion (BIC) and likelihood-ratio tests. Significant pairwise comparisons were explored using the ‘emmeans’ function (R package ‘emmeans’; https://CRAN.R-project.org/package=emmeans). For fruit-based oviposition choice (step 2), the proportion of eggs laid on each fruit puree was analysed using a binomial generalised linear model (GLM) with a logit link (R package ‘lme4’; Bates et al., 2015). Temperature and *Wolbachia* infection were included as the continuous predictor and categorical fixed effect, respectively. For temperature-based oviposition choice (step 3), the proportion of eggs laid at each temperature was analysed using quasibinomial GLM to account for overdispersion (R package ‘lme4’; Bates et al., 2015) with temperature and *Wolbachia* infection as the continuous predictor and categorical fixed effect, respectively. For fruit and temperature-based oviposition choice (step 4), preference was analysed using a baseline-category multinomial logistic model fitted with the ‘vglm’ function (R package VGAM; Yee, 2008). *Rubus idaeus* was excluded automatically because it received zero eggs, and *R. nigrum* was set as the reference category. Statistical significance was assessed using Wald χ² tests (‘Anova’ function of R package ‘car; Fox and Weisberg, 2019). All analyses were performed in R version 4.1.3 (https://www.r-project.org/).

## RESULTS

### Step 1: Performance in no choice experiments

The aim of this step was to identify the independent and interactive effects of temperature, fruit and *Wolbachia* infection on the performance of *D. suzukii* to produce quantitative predictions about the optimality of the decision making in the following steps. Hereafter, we present the results of number of eggs, offspring survival, development time and mass of offspring successively. Sample sizes are provided in Table S1.

#### Number of eggs

**Fig. 2.**
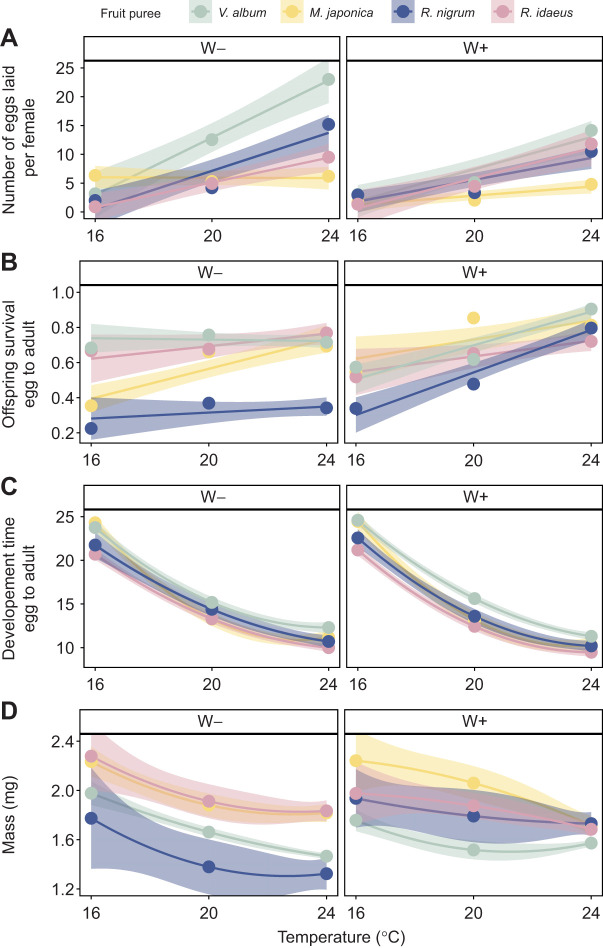
**Performance of *Drosophila suzukii* (step 1).** Mean number of eggs laid (A), egg-to-adult survival (B) and mean development time (days; C) and offspring mass (mg; D) on each fruit puree (*Viscum album*, *Mahonia japonica*, *Ribes nigrum* and *Rubus idaeus*) at three temperatures (i.e. 16, 20 and 24°C) for *Wolbachia*-uninfected (W–) and -infected (W+) individuals. Each combination was tested with 20 females (*N*=20) except for survival and mass (*N*=11–20 and *N*=4–20; see Table S1 for details). Solid lines are linear regressions for each fruit puree (GLMM; see Materials and Methods for details) and bands represent 95% confidence intervals.

The total number of eggs laid per female was influenced by fruit puree (GLMM: *P*<0.0001), temperature (*P*<0.0001), *Wolbachia* infection (*P*<0.0001) and their interactions (Fig. 2A; Table S2A). Overall, the highest number of eggs was laid on *V. album*. The number of eggs laid increased with temperature. The difference in number of eggs laid in each fruit also increased with temperature: at 16°C, the difference in average number of eggs laid between fruit purees was approximately five eggs (*R. idaeus*: mean±s.d.=0.90±0.22; *M. japonica*: 6.35±1.20), whereas at 24°C the difference was approximately 17 eggs (*M. japonica*: 6.20±1.15; *V. album*: 23.00±3.20) for W– females. The same pattern, even if less pronounced, was found in W+ females with a difference between fruit purees of two eggs at 16°C and a difference of 10 eggs at 24°C.

#### Offspring survival

Egg-to-adult survival of offspring was influenced by fruit puree (GLMM: *P*<0.0001), temperature (*P*<0.0001), *Wolbachia* infection (*P*=0.0001) and their interactions (Fig. 2B; Table S2B). Overall, survival was lowest on *R. nigrum*. Survival increased with temperature but, contrary to the number of eggs laid, higher temperature reduced the differences between fruit. *Wolbachia*-infected offspring had a higher survival compared with W– offspring (0.73±0.45 and 0.61±0.49, respectively). The most striking effect of *Wolbachia* was found on *R. nigrum*, as the survival of offspring that developed at 24°C was two-fold higher when infected (W–: 0.34±0.48; W+: 0.80±0.40; *post hoc* test: *P<*0.0001). A similar effect of *Wolbachia* on survival was obtained on *M. japonica* at 16°C (W–: 0.35±0.48; W+: 0.55±0.51; *post hoc* test: *P*=0.0481).

#### Development time

Egg-to-adult development time was influenced by fruit puree (GLMM: *P*<0.0001), temperature (linear and quadratic terms both *P*<0.0001), *Wolbachia* infection (*P=*0.0018) and their interactions (Fig. 2C; Table S2C). Overall, development of *D. suzukii* was faster at higher temperatures (*P*<0.0001). At all temperatures, development time was the longest on *V. album*, followed by *M. japonica*, whereas it was the shortest on both *R. nigrum* and *R. idaeus*. Overall, W+ individuals exhibited a shorter development time compared with W– individuals (14.52±5.31 and 15.03±4.98 days, respectively; *P=*0.0018). Temperature had the strongest effect, development time being two times faster at 24°C compared with 16°C, resulting in a difference of approximately 1 week. On the contrary, the differences between fruit were only approximately 1 to 4 days.

#### Mass

Female offspring were significantly heavier than males (1.67±0.01 and 1.35±0.01 mg, respectively; GLMM: *P<*0.0001) and we only considered females for further analyses. Female offspring mass was influenced by fruit puree (GLMM: *P*<0.0001), temperature (linear and quadratic terms *P*=0.0005 and *P*=0.0047, respectively), *Wolbachia* infection (*P*<0.0019) and their interactions (Fig. 2D; Table S2D). The heaviest females developed on *R. idaeus* and *M. japonica*. The mean offspring mass decreased with temperature (16°C: 2.02±0.03 mg; 20°C: 1.74±0.02 mg; 24°C: 1.60±0.01 mg). There was no overall significant difference in mass between W+ and W– females (1.69±0.01 and 1.65±0.01 mg, respectively; *P*=0.3433). However, at 24°C, females that developed on *R. nigrum* and *V. album* were heavier when infected by *Wolbachia* (*P*<0.0001 and *P*=0.0010, respectively).

Overall, the performance of *D. suzukii* was considered highest at 24°C, as this temperature maximised the number of eggs, offspring survival and development rate, particularly on *V. album* and *R. idaeus* fruit purees, which were most commonly associated with highest values for each of these traits.

### Step 2: Nutrition-based oviposition choice

**Fig. 3.**
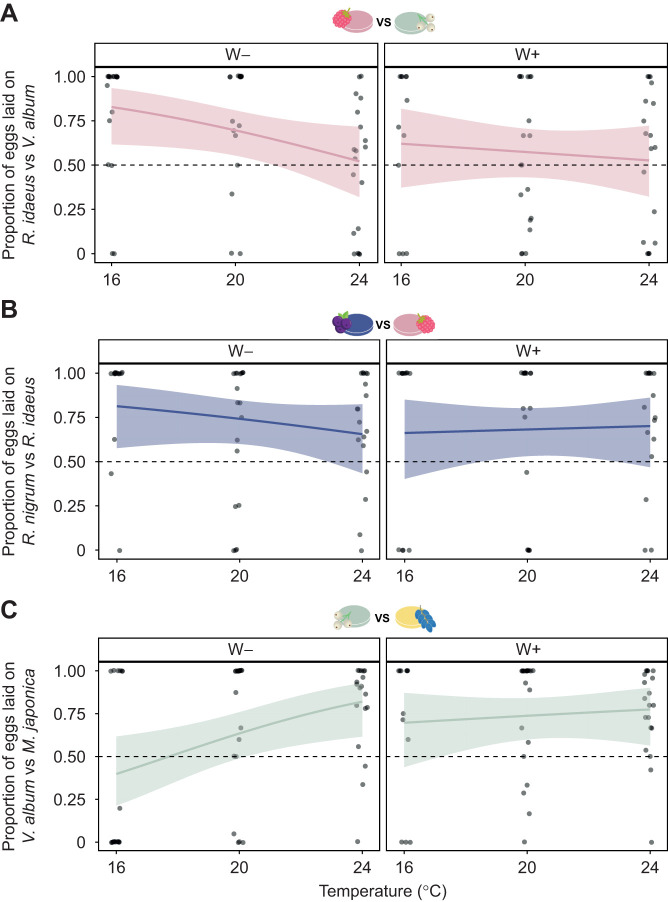
**Simple decision making – fruit (step 2).** Mean proportion of eggs laid by *Wolbachia*-infected (W+) and -uninfected (W–) *D. suzukii* females between (A) *R. nigrum* and *R. idaeus*, (B) *R. idaeus* and *V. album* or (C) *V. album* and *M. japonica* at each temperature (i.e. 16, 20 and 24°C). Each combination was tested with 20 females (*N*=20) except for W– females on *R. idaeus* versus *R. nigrum* at 16°C and W– and W+ females on *V. album* versus *M. japonica* at 16°C (*N*=19). Points represent the proportion of eggs laid by individual females, solid lines are linear regressions (GLM with binomial distribution and logit link; Table S4) and bands represent 95% confidence intervals.

In this step, females were exposed to a binary choice between fruit purees (i.e. *M. japonica* versus *V. album*, *R. nigrum* versus *R. idaeus*, and *R. idaeus* versus *V. album*) to assess whether their decision was optimal in terms of nutrition. Based on step 1, females were expected to lay an even number of eggs on *V. album* and *R. idaeus* (considered the two best fruit), and a higher number of eggs on these fruits when compared with *M. japonica* or *R. nigrum*. The results partially matched these hypotheses. The mean percentages of eggs laid for each combination of fruit are in Table S3. When exposed to *R. idaeus* and *V. album*, W– females laid more eggs on *R. idaeus* (GLM: *P*<0.0001; Table S4A; Fig. 3A) but the preference decreased with temperature (*P*<0.0001; Table S4A; Fig. 3A), whereas *Wolbachia* suppressed the preference (*P*<0.0001: Table S4A; Fig. 3A). Females exposed to *R. nigrum* and *R. idaeus* did not show any preference (GLM: *P*=0.3188; Table S4B; Fig. 3B), with temperature having no effect on this pattern (*P*=0.9719; Table S4B), but *Wolbachia* increasing the probability of oviposition in *R. ideaus* (*P*=0.0452; Table S4B). When exposed to *V. album* and *M. japonica*, females laid more eggs on *M. japonica* at cold temperature (GLM: *P*<0.0001; Table S4C; Fig. 3C), but temperature increased preference for *V. album* (*P*<0.0001; Table S4C; Fig. 3C), as did *Wolbachia* (interaction temperature×*Wolbachia*: *P*=0.0006; Table S4C; Fig. 3C).

### Step 3: Temperature-based oviposition choices

In this step, females were exposed to a binary temperature choice with two cylinders of the same fruit puree, one at 16°C and one at 24°C. The four fruit purees were tested, and according to step 1, 24°C was expected to be chosen over 16°C regardless of the fruit puree. Indeed, females consistently laid more eggs in the fruit purees kept at 24°C (GLM: *P*=0.0019; Table 1; Table S5). Overall, only few eggs were laid at 16°C (e.g. 5 and 12 eggs by W– and W+ females in *V. album*, respectively; 1 egg by W– females on *R. idaeus*). *Wolbachia* had no detectable effect on temperature preference (*P*=0.5410; Table S5).

**Table 1.** Simple decision making – temperature (step 3)

	Mean percentage of eggs laid			
	W–		W+	
Fruit puree	16°C	24°C	16°C	24°C
*Mahonia japonica*	26	74	0	0
*Rubus idaeus*	2.1	97.9	0	100
*Ribes nigrum*	2.5	97.5	20	80
*Viscum album*	18.8	81.2	18.9	81.1

Mean percentage of eggs laid by *Wolbachia*-uninfected (W–) and -infected (W+) *Drosophila suzukii* females during the temperature-based oviposition choice on the same fruit puree (i.e. *M. japonica*, *R. idaeus*, *R. nigrum* or *V. album*). Each combination was tested with 10 females (*N*=10) except for W– females on *V. album* (*N*=11) and on *R. ideaus* (*N*=12). Females oviposit preferentially on warm substrates (GLM: *P*=0.0019; Table S4) and *Wolbachia* had no detectable effect on temperature preference (*P*=0.5410; Table S4).

### Step 4: Optimal decision making

In this step, the aim was to decipher the strategy of *D. suzukii* females regarding oviposition decision making under complex situations mixing both temperature and nutrition choices. Females were exposed simultaneously to the four fruit purees, with the ones considered optimal based on step 1 (i.e. *R. idaeus* and *V. album*) cooled at 16°C (suboptimal temperature) and the ones considered suboptimal (i.e. *R. idaeus* and *V. album*) warmed at 24°C (optimal temperature).

**Fig. 4.**
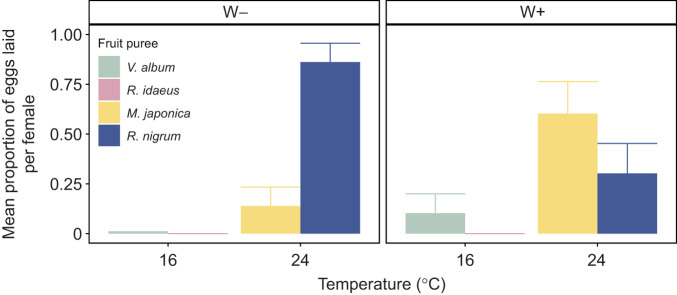
**Complex decision making (step 4).** Mean proportion of eggs laid by *Wolbachia*-uninfected (W–; *N*=11) and -infected (W+; *N*=10) *D. suzukii* females when fruit purees are crossed with temperature: *V. album* and *R. idaeus* at 16°C, and *M. japonica* and *R. nigrum* at 24°C. Females oviposited preferentially on warm substrates (multinomial regression with *R. nigrum* used as reference: *P*<0.0001) and *Wolbachia* infection altered the preference (multinomial regression: *P*<0.0001) (Table S6).

The oviposition preference of females varied significantly in this design (Fig. 4; Table S6). Both W– and W+ females showed a significant preference for suboptimal fruit purees kept at 24°C (i.e. *M. japonica* and *R. nigrum*) compared with optimal fruit purees kept at 16°C (i.e. *R. idaeus* and *V. album*). Only a few eggs were laid in *V. album* at 16°C (W–: 0.01±0.02 eggs; W+: 0.10±0.32 eggs) and none in *R. idaeus*. Overall, oviposition was strongly biased toward *R. nigrum* in W– females, with much lower probabilities for *M. japonica* and especially *V. album*. *Wolbachia* infection significantly altered this pattern (MLN: *P*<0.0001; Table S6), increasing the relative probability of oviposition on both *V. album* and *M. japonica* compared with *R. nigrum*, therefore reducing the strong preference for *R. nigrum* observed in W– females (Fig. 4).

## DISCUSSION

The PPH predicts that females maximise their fitness by laying eggs preferentially on substrates that maximise their offspring performance (Gripenberg et al., 2010). Most studies investigating this hypothesis considered one influential variable at a time, typically neglecting abiotic factors such as substrate temperature. Using the invasive fruit fly *D. suzukii*, we found clear evidence for the PPH, especially under complex choice scenarios in which temperature is involved. Here, we discuss (1) the influence of temperature and fruit on the performance of *D. suzukii*, (2) how *D. suzukii* females make optimal decisions for the performance of their offspring, (3) how the presence of *Wolbachia* influences performance and oviposition choice, and (4) what these results imply under climate change.

### Influence of temperature and fruit on *D. suzukii* performance

*Drosophila suzukii* performed better at 24°C, with more eggs, higher offspring survival and faster development compared with 16°C and 20°C, which was in accordance with previous studies (e.g. Raynaud-Berton et al., 2024). High temperature commonly increases female activity, including oviposition rate (Shaw et al., 2018). Interestingly, however, higher temperature did not increase oviposition on *M. japonica*, which was consistently low, indicative of lower host acceptance and egg withholding (Yang et al., 2008; Soto et al., 2012). The shared coevolutionary history between *D. suzukii* and *M. japonica* (same native range) could have allowed the development of defense strategies to repel oviposition, as is the case between most phytophagous insects and their host plants (Bruce, 2015).

Fruit puree influenced the number of eggs laid, offspring survival, development time and mass, with *R. idaeus* and *V. album* ranking better overall than *M. japonica* and *R. nigrum*, as expected from previous studies (Poyet et al., 2015; Olazcuaga et al., 2019; Deconninck et al., 2025a; Larges et al., 2025). The number of eggs laid by *D. suzukii* females was highly variable between fruit and could have been influenced by olfactory cues (Little et al., 2020; Tait et al., 2020; Akutsu and Matsuo, 2022) and egg withholding strategy as mentioned above (Yang et al., 2008; Soto et al., 2012).

Nutritional composition differences between fruit are likely to explain the differences in larval survival, development time and mass. The intermediate protein:carbohydrate (P:C) ratios in *V. album* (0.44) and *R. idaeus* (0.57) compared with *R. nigrum* (0.31; https://ciqual.anses.fr/; Larges et al., 2025) are close to the optimal 0.50 that increases larval performance in *D. suzukii* (Silva-Soares et al., 2017), with higher survival and faster development resulting in larger flies (Silva-Soares et al., 2017; Rendon et al., 2018; Larges et al., 2025).

The presence of toxic compounds might have also impaired development. *Ribes nigrum* berries contain important concentrations of phenolics, flavonoids and tannins (Paunović et al., 2022) that can impair development and survival in insects (Oparaeke and Daria, 2005; Chrzanowski et al., 2012) and could explain the lower offspring survival and mass on this fruit. Interestingly, although *V. album* also contains toxic compounds (e.g. lectin; Gaidamashvili et al., 2012), offspring performance was not impaired, consistent with other studies (Deconninck et al., 2024a; Larges et al., 2025), which might indicate that *D. suzukii* is resistant to lectin (Pal Mahadevan et al., 2025).

### Do *D. suzukii* females make the right oviposition choice?

According to the PPH, females should select oviposition sites that offer optimal performance for their offspring (Gripenberg et al., 2010). Based on step 1 and existing literature (Poyet et al., 2015; Olazcuaga et al., 2019; Larges et al., 2025), *D. suzukii* females were expected to prefer ovipositing on *V. album* and *R. idaeus* fruit purees and at higher substrate temperature.

In terms of fruit choice (step 2), the results aligned with the PPH when females had to choose between *V. album* and *M. japonica*, but not between *R. nigrum* and *R. idaeus*, where females oviposited evenly. A strong attraction for *R. nigrum* despite low performance was already noticed in a previous study (Olazcuaga et al., 2019). It was initially attributed to the high phosphorus content of berries, but another recent study suggests that inorganic phosphate is not the main driver of preference, and that phospholipids or other molecules correlated with phosphorus could explain this attraction (Olazcuaga et al., 2023). An alternative explanation could be the presence of compounds in *R. nigrum* that coincidently repel Drosophilidae parasitoids, such as in *Atropa belladonna* (Poyet et al., 2017). The host plants of *D. suzukii* that coexist in natural systems can differ in their attractiveness to or protection against parasitoids and influence the level of parasitisation of the fly (Viciriuc et al., 2025). The consideration of the third trophic level could partly explain the apparent deviations from the PPH. When females had to choose between *V. album* and *R. idaeus*, preference for *R. idaeus* was observed at low temperature, but as temperature increased, the eggs were distributed more evenly. The preference for *R. idaeus* at cold temperature aligns with the PPH, as even if survival did not differ between both fruit, *R. idaeus* allowed a shorter development, resulting in larger flies compared with *V. album*. The absence of choice at higher temperature could reflect the reduction of the contrast between fruit in terms of performance when temperature increases. During the oviposition selection process, *D. suzukii* females are influenced by numerous olfactory, visual (e.g. colour, size, shape) and tactile (e.g. texture, hardness, firmness) cues (Little et al., 2020; Akutsu and Matsuo, 2022), as well as the presence of specific compounds (e.g. sugar; Ulmer et al., 2022) or pH levels (Bühlmann and Gossner, 2022). Surprisingly, P:C ratios do not influence preference (Silva-Soares et al., 2017). Conflicts between female nutritional needs and optimal oviposition site can also occur (Lihoreau et al., 2016). Identifying the factors that contribute the most to fruit or substrate selection in *D. suzukii* could facilitate the understanding of the discrepancies found between studies on PPH in this species (Poyet et al., 2015; Young et al., 2018; Olazcuaga et al., 2019; Larges et al., 2025).

In terms of substrate temperature choice (step 3), *D. suzukii* preferentially oviposited at 24°C, aligning with the PPH. The temperature of the oviposition site can provide reliable cues about the thermal conditions for larval development, especially when larval motility is limited (Silva-Soares et al., 2017; Reyes-Ramírez et al., 2023). In the peach moth *Grapholita* (*Cydia*) *molesta*, females preferentially oviposited at 30°C, a temperature that accelerates development, maximises offspring survival and results in higher pupal mass (Notter-Hausmann and Dorn, 2010). However, sometimes females appear to choose less favourable sites (e.g. Rausher, 1979; Stoeckli et al., 2008), and this could be explained in part by the experimental design. Our TLS (or any thermal gradient such as in Notter-Hausmann and Dorn, 2010) allows for the evaluation of the direct effect of the substrate temperature independently of the temperature experienced by the female. Given that larvae and adults have contrasting thermal needs and tolerance (Sinclair et al., 2016; Raynaud-Berton et al., 2024), there is likely a conflict in the female decision between the physiological effect of substrate temperature on its offspring and the optimal body temperature it can obtain in the microenvironment near oviposition substrate, as the natural thermal landscape can be immensely heterogeneous over small scales for insects (Pincebourde and Woods, 2020; Pincebourde et al., 2021). This property can be key to understanding the relationship between female preference and offspring performance. In our study, 24°C is supposed to be optimal for both the female and its progeny (compared with 16°C). This means that we cannot entirely decipher whether females laid more eggs in the warmer substrate because they are actively choosing the warmer temperature for their offspring, or because they select the warm temperature for themselves and oviposit were they sit. However, the second option is unlikely as preliminary data indicate that fertile females of *D. suzukii* have thermal preference around 21°C (G. Deconninck, personal observation). Future studies on decision making could further explore decisions when there is a mismatch between the female needs and the optimal temperature for offspring (e.g. Neu et al., 2021).

Under complex situations (combination of suboptimal fruit at optimal substrate temperature and vice versa; step 4), females preferentially oviposited on suboptimal fruit purees kept at optimal temperature, indicating that females prioritised temperature over nutrition during oviposition choice. These results suggest that the PPH is supported particularly in this multifactorial context as temperature induced larger changes in offspring performance than the fruit purees. For instance, differences in development time between fruit were approximately 4 days whereas differences between 16°C and 24°C were approximately 10 days. Interestingly, females oviposited some eggs in *V. album* at 16°C. This could be a bet-hedging strategy (Hopper, 1999), because spreading eggs across different microclimatic and nutritional environments could maximise the survival chances of at least some of the offspring (Pincebourde et al., 2007).

### *Wolbachia* influences performance and oviposition choice

*Wolbachia* influenced both performance and oviposition choice in *D. suzukii*. Overall, W+ females laid fewer eggs compared with W– females, but this reduction was offset by increased survival rates, faster development and greater mass of offspring, in accordance with previous studies (Hamm et al., 2014; Mazzetto et al., 2015; Deconninck et al., 2024c). *Wolbachia* infection also changed oviposition preference between fruit purees (step 2), but not in the temperature choice (step 3). Although *Wolbachia* is known to impact thermal preference (Truitt et al., 2019; Hague et al., 2021), its influence on oviposition choice has rarely been explored (but see Zchori-Fein et al., 2001; Vala et al., 2004; Wiwatanaratanabutr et al., 2010). In complex decision scenarios (step 4), and in some instances for temperature and fruit choices, *Wolbachia*-infected females seemed to distribute their eggs more evenly. This result is in accordance with previous studies on parasitoid wasps suggesting that *Wolbachia* infection impairs decision making by reducing the ability of females to discriminate between low and high quality hosts or between already parasitized or unparazitized hosts (Kishani Farahani et al., 2015; Abrun et al., 2021). The infection by *Wolbachia* also seems to reduce the differences in terms of performance between fruit (as shown by Deconninck et al., 2024c), which would reduce the need to discriminate between them and could lead to bet-hedging. More research is needed to understand the complex effect of *Wolbachia* on host selection in *D. suzukii*, integrating both thermal preference and oviposition behaviour.

### Ecological implications in an era of global changes

*Drosophila suzukii* females consistently prioritised thermally optimal substrates that, overall, increased their offspring performance. As nutrition was not the strongest determinant of oviposition choice, behavioural decisions in this species appear primarily constrained by thermal conditions. Climate change reshapes the availability of suitable microhabitats (Kemppinen et al., 2024; Kerr et al., 2025) and modifies the distribution and phenology of host plants (Parmesan, 2006; Bellard et al., 2012). Climate warming is likely to facilitate the spread of *D. suzukii* into cooler areas (Asplen et al., 2015; Wiman et al., 2016) and potentially increase the number of suitable host plants (development of *D. suzukii* could be made possible in warmer fruit compared with cold fruit). Climate-driven shifts in the distribution and fruiting phenology of key host plants, including non-native ornamental species such as *Elaeagnus* × *submacrophylla*, *M. japonica* or *Aucuba japonica* (Deconninck et al., 2025a; Larges et al., 2025) may further broaden the mosaic of oviposition opportunities encountered by females. We predict that warming combined with a broad range of fruit resources should reinforce the performance of this fruit fly in early spring, when populations start to build up after overwintering. However, one caveat concerns the lack of knowledge on the thermoregulation behaviour of this fly (but see Deconninck et al., 2025b for an example on *D. melanogaster*).

Moreover, infection with *Wolbachia* improved the overall performance of *D. suzukii*, as in Deconninck et al. (2024c), while also leading to a more even distribution of eggs in available substrates. Symbionts are generally known to modify host thermal tolerance, reproduction and ecological interactions (Frago et al., 2012; Corbin et al., 2017), with implications for niche extent and adaptation to new conditions (Lemoine et al., 2020). As such, *Wolbachia* could influence how *D. suzukii* responds to novel combinations of temperature and host availability under future climates. In the field, the prevalence of *Wolbachia* in *D. suzukii* populations can shift dramatically between generations (McPherson et al., 2023), although the underlying reasons remain to be elucidated. According to our results, this would have implications on the host plants chosen by the fly to oviposit from one generation to the next and therefore on the overall fitness of the populations.

Overall, our findings suggest that temperature–host–symbiont interactions may shape the future distribution, invasion potential and economic impact of *D. suzukii*, and more broadly highlight the need to study behavioural decisions under conflicting environmental constraints to understand how behavioural flexibility contributes to population persistence under global change.

### Conclusions

Our study highlights the dominant role of temperature in shaping oviposition choices in *D. suzukii*, showing that females prioritise thermal conditions over the nutritional quality of the substrate. This finding raises intriguing questions about other polyphagous insects: do they exhibit a similar optimal decision making between temperature and nutritional quality when selecting oviposition sites? Comparative studies could reveal similar patterns or fundamental differences in the egg-laying strategies of species differing in their invasion abilities or feeding behaviour. Moreover, we showed for the first time that infection by *Wolbachia* influences the oviposition choice in *D. suzukii*. How host–symbiont interactions influence oviposition behaviour under complex, more natural environmental situations remains to be explored. More generally, the oviposition choices of ectotherms based on substrate temperatures can be understood in the light of the microclimatic conditions at a rather fine spatial scale, but they remain unknown for most ectotherms. Experimental designs with such complex scenarios involving mixing optimal and suboptimal cues should be expanded more largely, especially to understand how organisms respond to environmental change that does not affect all factors equally.

## Supplementary Material

10.1242/jexbio.251743_sup1 Supplementary information
