# Supplementary material for: Temperature overrides nutritional cues for optimal oviposition decision in a polyphagous invasive insect
Source: J Exp Biol. 2026 Feb 12;229(3):jeb251743. doi: 10.1242/jeb.251743 (PMC12968785; doi:10.1242/jeb.251743)
Supplement: Supplementary information [file jexbio-229-251743-s1.pdf]

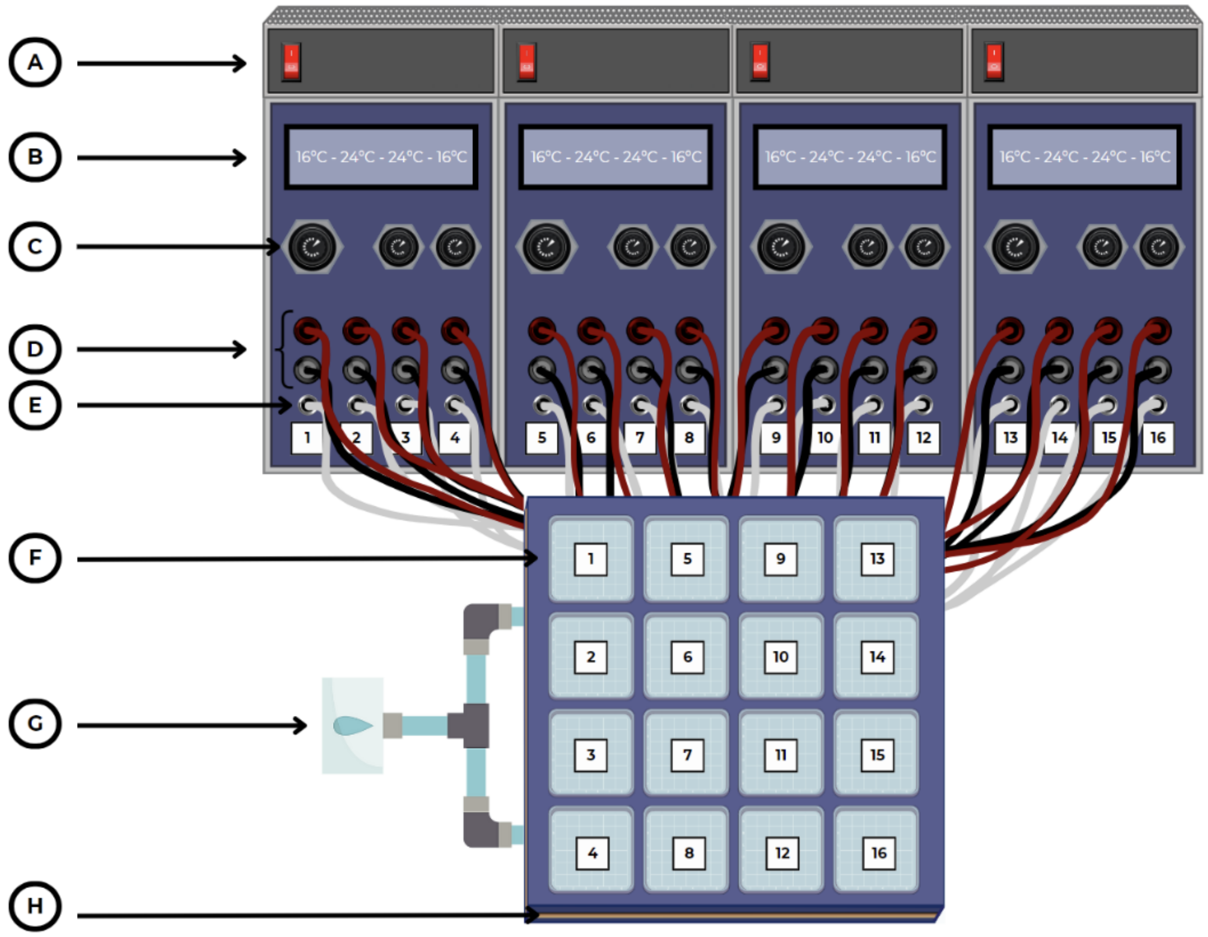

Fig. S1. Thermal landscape simulator (TLS) that allows the measurement of the trophic and thermal preference of females. Numbers from 1 to 16 indicate the number of Peltiers and aluminium plates associated with each temperature controller. Letters from A to G represent the different elements of the thermal landscape: A. Power supply, B. Temperature controllers, C. Setting buttons, D. Electric cables supplying power to each Peltier, E. Temperature probes, F. Aluminium plates, G. Water-cooling system, H. Peltier.

Journal of Experimental Biology • Supplementary information

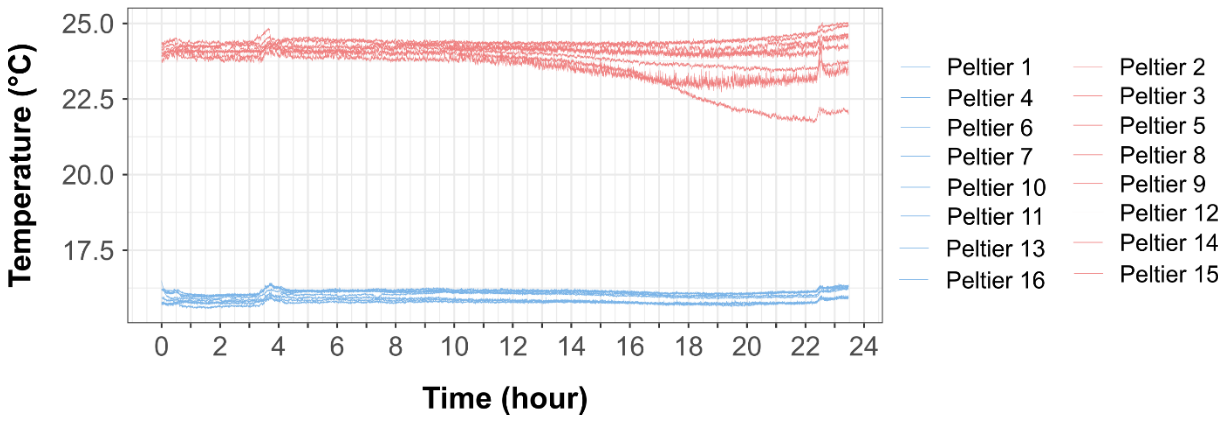

Fig. S2. Temperature calibration for Peltier units 1 to 16 took approximately 24 hours. The blue and red colours represent temperature variations. Blue: Peltier unit set at 16 °C, red: Peltier unit set to 24 °C.

Table S1. Sample size for Step 1 – Performancein no-choice experiments.

			Experiments			
**Fruit puree**	**Temperature**	***Wolbachia***	**Number of eggs laid**	**Survival**	**Development time**	**Female mass**
M. japonica	16	W-	20	19	20	11
		W+	20	13	20	5
	20	W-	20	19	20	16
		W+	20	16	20	11
	24	W-	20	19	20	16
		W+	20	20	20	16
R. idaeus	16	W-	20	11	20	7
		W+	20	17	20	4
	20	W-	20	18	20	11
		W+	20	18	20	11
	24	W-	20	19	20	16
		W+	20	18	20	17
R. nigrum	16	W-	20	15	20	4
		W+	20	16	20	6
	20	W-	20	20	20	10
		W+	20	17	20	8
	24	W-	20	19	20	13
		W+	20	20	20	19
V. album	16	W-	20	16	20	10
		W+	20	13	20	9
	20	W-	20	20	20	16
		W+	20	19	20	12
	24	W-	20	19	20	18
		W+	20	20	20	20

Journal of Experimental Biology • Supplementary information

Table S2. Summary of Step 1 results with (a) generalised linear mixed models (GLMM) on total number of eggs laid per female, (b) GLMM on offspring survival, (c) linear model (LM) on log-transformed egg-to-adult development time and (d) GLMM on female offspring mass (mg).

**Step 1 - Performance in no choice experiments**			
**(A) Total number of eggs laid per female**			
	Chisq	Df	*p*-value
Fruit puree (FP)	44.801	3	**1.02e-09**
Temperature (Temp)	202.833	1	**<2.2e-16**
Wolbachia (Wol)	16.453	1	**4.99e-05**
FP × Temp	34.860	3	**1.30e-07**
FP × Wol	17.645	3	**0.0005**
Temp × Wol	0.012	1	0.9127
FP × Temp × Wol	10.515	3	**0.0147**
**(B) Offspring survival**			
	Chisq	Df	*p*-value
Fruit puree (FP)	43.931	3	**1.56e-09**
Temperature (Temp)	28.226	1	**1.08e-07**
Wolbachia (Wol)	14.714	1	**0.0001**
FP × Temp	2.211	3	0.5298
FP × Wol	10.740	3	**0.0132**
Temp × Wol	6.905	1	**0.0086**
FP × Temp × Wol	6.546	3	0.0879
**(C) Mean egg-to-adult development time**			
	Chisq	Df	*p*-value
Fruit puree (FP)	181.323	3	**<2.2e-16**
Temperature (Temp)	254.149	1	**<2.2e-16**
Temperature^2 (Temp^2)	151.859	1	**<2.2e-16**
Wolbachia (Wol)	9.792	1	**0.0018**
FP × Temp	23.295	3	**3.51e-05**
FP × Temp^2	22.216	3	**5.88e-05**
FP × Wol	1.114	3	0.7738
Temp × Wol	0.003	1	0.9534

Journal of Experimental Biology • Supplementary information

$Temp^2$ × Wol	0.024	1	0.8776
FP × Temp × Wol	5.011	3	0.1710
FP × $Temp^2$ × Wol	5.314	3	0.1502

(D) Female offspring mass (mg)

	Chiq	Df	*p*-value
Fruit puree (FP)	184.302	3	**<2.2e-16**
Temperature (Temp)	12.131	1	**0.0005**
$Temperature^2$ ($Temp^2$)	7.996	1	**0.0047**
Wolbachia (Wol)	9.643	1	**0.0019**
FP × Temp	0.684	3	0.8769
FP × $Temp^2$	0.741	3	0.8636
FP × Wol	83.480	3	**<2.2e-16**
Temp × Wol	1.640	1	0.2003
$Temp^2$ × Wol	1.379	1	0.2402
FP × Temp × Wol	5.848	3	0.1192
FP × $Temp^2$ × Wol	6.928	3	0.0742

Chisq = Chi-Square; Df = Degrees of freedom. Significant *p*-values ($p < 0.05$) are in bold.

Journal of Experimental Biology • Supplementary information

Table S3. Fruit-based oviposition choices (Step 2). Mean percentage of eggs laid by W– and W+ *D. suzukii* females during the fruit-based oviposition choice with (a) *R. idaeus* versus *R. nigrum*, (b) *R. idaeus* versus *V. album* and (c) *V. album* versus *M. japonica.*

**Step 2 - Fruit-based oviposition choices**				
**(A) *R. idaeus* vs *R. nigrum* - Mean percentage of eggs laid by females**				
	W–		W+	
	V. album	*R. idaeus*	*V. album*	*R. idaeus*
16 °C	20.6	79.4	35.4	64.6
20 °C	24.1	75.9	45.8	54.2
24 °C	50.9	49.1	45.7	54.3
**(B) *R. idaeus* vs *V. album* - Mean percentage of eggs laid by females**				
	W–		W+	
	R. nigrum	*R. idaeus*	*R. nigrum*	*R. idaeus*
16 °C	86.1	13.9	66.7	33.3
20 °C	66.7	33.3	67.4	32.6
24 °C	69.2	30.8	70.5	29.5
**(C) *V. album* vs *M. japonica* - Mean percentage of eggs laid by females**				
	W–		W+	
	V. album	*M. japonica*	*V. album*	*M. japonica*
16 °C	36.5	63.5	67.2	32.8
20 °C	69.4	30.6	76.8	23.2
24 °C	78.9	21.1	75.9	24.1

Journal of Experimental Biology • Supplementary information

Table S4. Summary of Step 2 results with GLM on mean proportion of eggs laid by females on (a) *R. idaeus* versus *V. album*, (b) *R. idaeus* versus *R. nigrum* and (c) *V. album* versus *M. japonica.*

**Step 2 - Fruit-based oviposition choices**				
**(A) *R. idaeus* vs *V. album* - Mean proportion of eggs laid by females**				
	Estimate	sd	*z*-value	*p-value*
Intercept	7.8056	0.8988	8.684	**<2e-16**
Temperature (Temp)	-0.3365	0.0396	-8.496	**<2e-16**
Wolbachia (Wol)	-6.6717	1.1631	-5.736	**9.67e-09**
Temp × Wol	0.3055	0.0513	5.956	**2.59e-09**
**(B) *R. idaeus* vs *R. nigrum* - Mean proportion of eggs laid by females**				
	Estimate	sd	*z*-value	*p-value*
Intercept	0.9595	0.9624	0.997	0.3188
Temperature (Temp)	-0.0015	0.0435	-0.035	0.9719
Wolbachia (Wol)	-2.7504	1.3731	-2.003	**0.0452**
Temp × Wol	0.0983	0.0616	1.594	0.1109
**(C) *V. album* vs *M. japonica* - Mean proportion of eggs laid by females**				
	Estimate	sd	*z*-value	*p-value*
Intercept	-5.1351	0.8297	-6.189	**6.04e-10**
Temperature (Temp)	0.2737	0.0380	7.212	**5.53e-13**
Wolbachia (Wol)	4.5969	1.1707	3.927	**8.62e-05**
Temp × Wol	-0.1847	0.0535	-3.455	**0.0006**

Significant *p*-values (p <0.05) are in bold.

Table S5. Summary of Step 3 results with GLM on mean proportion of eggs laid by W– and W+ *D. suzukii* females during the temperature-based oviposition choice on the same fruit puree (*i.e., M. japonica, R. idaeus, R. nigrum* or *V. album*).

**Step 3 - Temperature-based oviposition choices**				
	Estimate	sd	*z*-value	*p-value*
Intercept (*V. album*)	2.0891	0.6459	3.235	**0.0019**
M. japonica	-0.9905	0.9833	-1.007	0.3174
R. nigrum	1.2817	0.9661	1.327	0.1891
R. idaeus	2.8197	1.5065	1.872	0.0656
Wolbachia	-0.4636	0.7546	-0.614	0.5410

Significant *p*-values (p <0.05) are in bold.

Table S6. Summary of Step 4 results with multinomial regression (MNL) on oviposition choice between suboptimal fruit purees kept at 24 °C (*i.e.*, *M. japonica* and *R. nigrum*), compared to optimal fruit purees kept at 16 °C (*i.e.*, *R. idaeus* and *V. album*) with *R. nigrum* used as reference.

**Step 4 - Temperature and fruit-based oviposition choices**								
	V. album vs *R. nigrum*				*M. japonica* vs *R. nigrum*			
Contrast (log-odds)	Estimate	sd	*z*-value	*p-value*	Estimate	sd	*z*-value	*p-value*
Intercept (W–)	-4.9345	1.0036	-4.917	**8.79e-07**	-1.9900	0.2446	-8.136	**4.08e-16**
Wol effect	2.4496	1.4458	1.694	0.0902	2.5962	0.4343	5.978	**2.26e-09**

Note: *R. idaeus* was excluded from the model as it received zero eggs.Significant *p*-values (p <0.05) are in bold.
